# Building a Professional Identity and an Academic Career Track in Translational Medicine

**DOI:** 10.3389/fmed.2019.00151

**Published:** 2019-07-03

**Authors:** Sabine J. van Dijk, Andrea A. Domenighetti, Natalia Gomez-Ospina, Patricia Hunter, Caroline A. Lindemans, Veerle Melotte, Annemarie M. C. van Rossum, Norman D. Rosenblum

**Affiliations:** ^1^Department of Pharmacology, University of California, Davis, Davis, CA, United States; ^2^The Shirley Ryan AbilityLab, Chicago, IL, United States; ^3^Department of Physical Medicine and Rehabilitation, Northwestern University, Chicago, IL, United States; ^4^Division of Medical Genetics, Department of Pediatrics, Stanford University, Stanford, CA, United States; ^5^UCL Great Ormond Street Institute of Child Health, University College of London, London, United Kingdom; ^6^University Medical Center Utrecht, Wilhelmina Children's Hospital (WKZ), Utrecht University, Utrecht, Netherlands; ^7^Princess Maxima Center for Pediatric Oncology, Utrecht, Netherlands; ^8^Department of Pathology, Maastricht University Medical Center, GROW School for Oncology and Developmental Biology, Maastricht, Netherlands; ^9^Department of Clinical Genetics, Erasmus MC University Medical Center, Rotterdam, Netherlands; ^10^Division of Infectious Diseases and Immunology, Department of Pediatrics, Erasmus MC University Medical Center-Sophia Children's Hospital, Rotterdam, Netherlands; ^11^Laboratory Medicine and Pathobiology, Departments of Paediatrics, Physiology, University of Toronto, Toronto, ON, Canada

**Keywords:** translational medicine, translational scientist, basic scientist, physician scientist, career track, biomedical sciences

## Abstract

Biomedical scientists aim to contribute to further understanding of disease pathogenesis and to develop new diagnostic and therapeutic tools that relieve disease burden. Yet the majority of biomedical scientists do not develop their academic career or professional identity as “translational scientists,” and are not actively involved in the continuum from scientific concept to development of new strategies that change medical practice. The collaborative nature of translational medicine and the lengthy process of bringing innovative findings from bench to bedside conflict with established pathways of building a career in academia. This collaborative approach also poses a problem for evaluating individual contributions and progress. The traditional evaluation of scientific success measured by the impact and number of publications and grants scientists achieve is inadequate when the product is a team effort that may take decades to complete. Further, where scientists are trained to be independent thinkers and to establish unique scientific niches, translational medicine depends on combining individual insights and strengths for the greater good. Training programs that are specifically geared to prepare scientists for a career in translational medicine are not widespread. In addition, the legal, regulatory, scientific and clinical infrastructure and support required for translational research is often underdeveloped in academic institutions and funding organizations, further discouraging the development and success of translational scientists in the academic setting. In this perspective we discuss challenges and potential solutions that could allow for physicians, physician scientists and basic scientists to develop a professional identity and a fruitful career in translational medicine.

## Building a Brighter Future for Translational Medicine

Biological and medical research has greatly excelled during the last 50 years with huge advances in understanding disease pathogenesis. Despite these advances, a large “translational gap” exists in linking promising scientific discoveries to therapeutic interventions that improve the outcome of disease (1, 2). The United States National Institute of Health's National Center for Advancing Translational Sciences (NCATS) defined translational science as “the process of turning observations in the laboratory, clinic, and community into interventions that improve the health of individuals and populations—from diagnostics and therapeutics to medical procedures and behavioral interventions.” The possibilities for translational scientists, whether they are physician scientists or basic scientists, to bring observations from the laboratory to the patient and vice versa has never been greater. Yet, important barriers exist, that prevent the participation of biomedical scientists in the field of translational medicine (Table 1). While physicians have been traditionally considered as the most likely candidates to drive the translational scientific field from the bench to bedside because of their direct interactions with patients, the number of physicians engaged in research has steadily decreased by almost 50% between 1985 and 2012 (3). Their participation has been inhibited by a variety of previously identified factors including - but not limited to - a lengthy training pathway, difficulties establishing a career in science alongside practicing medicine, and accumulation of extensive debt during training (4, 5). In turn, basic scientists face their own specific obstacles when pursuing a career in translational science, such as having limited access to patients and clinical data, and poor alignment of the academic career and promotion track with the timeline of translational research. In addition, there are common challenges faced by both physicians and basic scientists due to the unique position of translational science bridging academic and clinical environments. The field of translational science meets a great need to better connect science and medicine. However, the unique requirements that come with interdisciplinary and long-term research projects are vastly different from the traditional way by which we currently approach biomedical research. In this perspective we discuss challenges and potential solutions that could allow for physicians, physician scientists, and basic scientists to develop a professional identity and a fruitful career in translational medicine. We hope that this perspective will increase awareness of existing limitations in biomedical sciences and spark discussion about the significant shifts needed to move biomedical sciences toward a future in which new knowledge is optimally translated into improved medical care.

**Table 1:** Summary of major problem encountered by scientist pursuing a career in translational science and proposed solutions.

**Problem**	**Proposed solution**
The incentive and reward system within academia is poorly aligned with a career track in translational medicine	Academic institutions need to establish better evaluation processes for retention and promotion of their translational scientists: - Using metrics that recognize the wide range of potential contributions to translational medicine (clinical or social impact of the work, the degree of risk and innovation, the success in establishing multidisciplinary collaborations, reaching of specific milestones along the path of a project that could take long to complete, the successful launch of a product or clinical trial, and their involvement with and recognition by patient advocacy groups) - Clear guidelines on how individual contributions to large multidisciplinary efforts are evaluated and measured - Individualized or extended tenure clocks that are more proportional to the potential impact and better reflect the timelines of the work
The number of clinician scientists is declining due to longer training, high education costs and challenges combining clinical service and research	- Integrated training in biomedical science and clinical training, with a stipend and tuition allowance that helps reduce debt accumulation - Recognition that clinical care and research enrich each other, and clear expectations about the efforts made toward each appointment
The highly focused training of basic scientists with limited interdisciplinary or clinical exposure poorly prepares basic scientist for the collaborative nature of translational medicine	- Training in an interdisciplinary environment, including exposure to the clinic, will prepare basic scientists to excel in a collaborative translational research environment - Actively recruiting and encouragement of participation of basic scientists in existing advanced degree programs in Translational Research with courses and research projects focusing on team building, translational science, clinical research, epidemiology, translational therapeutics, entrepreneurial sciences, or biomedical informatics
The unique requirements of translational medicine, such as significant longer timelines, much larger number of lab members that can be funded with an average grant, larger overhead costs extending beyond the immediate duration and focus of the project, are not met by the current funding system	- Adjust time lines of grants, possibly by making funding for the next phase conditional on researching milestones - Divide increased costs, for example by requiring matching external funding from the research institution, an industry partner, or a patient advocacy group - Encourage young scientists to pursue a career in translational medicine by specifically providing funding for early-career scientists in their translational grants, and by removing restrictions such as a “time-since-PhD limit” on all personal fellowships in recognition that research does not always follow the anticipated timescale
Building an infrastructure to promote education and research in translational medicine is a resource-intensive enterprise	- Creation of and funding for centralized “hubs” or “cores” that are characterized by shared, multidisciplinary use of expensive laboratory equipment, data power and complex professional skills (e.g., genomics, imaging, flow cytometry, animal facilities, data, and biobanking) that are necessary to support large translational research projects - Create a collaborative and interdisciplinary environment where basic scientists and physician scientists, clinical personnel, patients and intellectual property officers interact regularly to support new thinking and to promote translational research

## Training Translational Scientists Should be Interdisciplinary and Collaborative

Clinical practice and research are two separate disciplines, and training as a scientist alongside obtaining a medical degree is arduous. Medical training has historically been long and expensive, and has lengthened over time. In addition, trends in MD program curricula are shifting toward more specialization, increased focus on health care systems and delivery of care, and include less scientific knowledge that informs pathobiology and treatment of disease. In response to these forces, the American National Institute of Health (NIH) created the Medical Scientist Training Program (MSTP) to train physician scientists (https://www.nigms.nih.gov/training/instpredoc/Pages/PredocOverview-MSTP.aspx). MSTP trainees follow an integrated training in biomedical science and clinical practice, and receive a stipend and tuition allowance that helps reduce debt accumulation. However, the program is very competitive and currently supports merely ~1,000 students. Programs with similar intent exist in Europe (e.g., AKO program, Maastricht University, The Netherlands), where a 4-years master program combines patient-oriented research and medical practice with the goal of translating the results of scientific research into the practice of patient care. However, like the MSTP, this program supports only a limited number of students.

Basic scientists receive highly specialized training, the nature of which is strongly influenced by their mentor and a small committee of professors with a similar research focus (6). As a result, graduate training is prone to have limited breadth and scope. Further, trainees are often discouraged to venture outside the scope of their thesis to explore independent projects as they are expected to work on a project of their mentor and to graduate within an acceptable time (Table 1). In contrast, success as a translational scientist requires training in collaborative and interdisciplinary environments (3). Not only is close collaboration within diverse teams of scientists and clinicians essential, translational medicine also involves participation from non-scientific stakeholders such as intellectual property officers, investors, patient advocacy groups, ethicists, and regulatory bodies. Although the strength of the team comes from the separate expertise of individuals, a common understanding of the entire process of translational medicine—including the diverse background, priorities and language of team members—is critical for successful teamwork. Diversity in a team increases creativity and likelihood of genuine innovation (7). Therefore, training in an interdisciplinary environment is essential for basic scientists to excel in translational research.

Considerable investment to develop and improve core programs supporting research career tracks in translational medicine has been made in the last two decades, and advanced degree programs in Translational Research have been established in many universities. Postdoctoral and pre-doctoral candidates participating in translational medicine programs are exposed to courses and research projects focusing on topics such as team building, translational science, clinical research, epidemiology, translational therapeutics, entrepreneurial sciences, and biomedical informatics. Interestingly, despite the fact that a large number of these academic programs are available to basic scientists and physicians alike, it seems that physician scientists are more likely to enroll in translational programs. For example, the large majority of registered students (~68%) for the Master of Science in Translational Research at the University of Pennsylvania were participants in an MD or an MD/PhD program, while PhD candidates formed only 7% of the student pool (As of August 2018, http://www.itmat.upenn.edu/mstr-alumni.html). Research institutes have an incredible opportunity to improve their translational medicine programs by actively recruiting more basic scientists to help create a more diverse population of researchers needed for a well-rounded translational medicine program.

## Career Evaluation and Advancement of Translational Scientists

Practicing physician scientists reported major challenges that limit the ability to engage in both clinical practice and research, with difficulty of balancing time between clinical, research, and teaching responsibilities as leading obstacle (5). Physician scientists are often affiliated with multiple clinical and research departments, each having different objectives and interests. Such shared appointments can easily lead to under appreciation of the efforts of the physician scientist when individual departments regard time spent in the other department as “lost time,” preventing the physician scientist from making a full contribution to their mission. Instead, clinical and research contributions should be evaluated in concert and departments should value the unique insights and experience a dual appointment can bring.

Performance and progress of basic scientists is mainly evaluated by the number of publications and authorship rankings, and by the amount of funding secured. The current review system and associated metrics result in pressure to publish promptly and frequently. These dynamics are important contributors to the increasingly recognized problems such as lack of reproducibility, invalidated data, avoidance of risky or team-oriented projects, and ultimately research waste (8–12). Furthermore, publication impact is quantified using parameters such as journal impact factor or H index, numbers that do not correlate with the quality or the social impact of the published work (13). Another limitation of this publication-driven environment is that individual contributions are impossible to discern and only first and last authors are fully recognized—although multiple journals now require a thorough description of individual contributions of each author in an attempt to give credit where due.

A large part of the solution resides in establishing evaluation and promotion processes that are more consistent with the goal of translational medicine: to improve human health. Because the nature of translational medicine is vastly different from current academic customs, academic leadership will first have to actively promote and reward a collaborative and translational scientific culture. Only when the value of translational science is well-embedded in the culture of universities, can evaluation criteria be developed that are better aligned with the requirements of translational science. Appraisal metrics should no longer rely primarily on number of publications and grants, but also recognize the wide range of potential contributions to translational medicine. A portfolio of “productivity” should be considered where not just the number of publications is included but also the potential clinical or social impact of the work, the degree of risk and innovation, successfully establishing multidisciplinary collaborations, reaching of predefined milestones within a continuing project, the launch of a product or clinical trial, and involvement with and recognition by patient advocacy groups. Institutions need to create clear guidelines that are well-disseminated on how individual contributions to large multidisciplinary efforts are evaluated and measured. These might include specific metrics for the different domains of the project: design, execution, and analysis in the basic science and clinical realm as evaluated by reviewers who can assess those individual contributions. Such a renewed evaluation process also requires a different composition of review committees, including interdisciplinary expertise from researchers, clinicians, and other healthcare professionals. Consideration should be given to individualized or extended tenure clocks that are more proportional to the potential impact of the research and better reflect the timelines of the work.

## Translational Science Requires a Different Funding System

A challenge faced by all translational scientists is that the unique requirements of translational medicine are not met by the current funding system. For example, the timeline of translational science is significantly longer than the duration of most funding cycles. In addition, a multidisciplinary translational team is much larger than the average number of lab members that can be funded with a grant. Lastly, the overhead costs in translational science are large and often extend beyond the immediate duration and focus of the project. An example is the development and maintenance of a biobank. Collecting well-preserved patient material over extended periods of time can be of crucial value to multiple translational research groups and projects, but in the current funding climate it is difficult to secure sufficient and long-term funding for such an endeavor. Research institutions could play a key role by creating an infrastructure that would allow long-term coverage of such shared resources, for example by creating a Translational Science Institute with a leadership that actively pursues funding for core facilities, e.g., through donors or collaborations with industry.

Securing independent funding has become more challenging, particularly for young scientists (14). The hypercompetitive funding situation has led to an academic environment that discourages collaboration, sharing of resources and open science practices, and the risk of pursuing projects that are either long, novel or difficult (15). The strict criteria and timescales for eligibility and outputs of early-career grants further encourage the pursuit of readily publishable research. In the UK, the Medical Research Council, Cancer Research UK and the Wellcome Trust have now removed their time-since-PhD limit on all personal fellowships in recognition that research does not always follow the anticipated timescale. Funding agencies could further encourage young scientists to pursue a career in translational medicine by specifically providing funding for early-career scientists within larger translational grants. Most beginning scientists have not built a large network yet, which makes it difficult to serve as the principal investigator and form a translational team needed to secure funding and make large project succeed.

Translational research is a long-term endeavor with uncertain outcome, and it is understandable that funding agencies have reservations committing large sums of money to such risky projects. A solution could be to make funding conditional. Continued funding could depend on performance and intermediate results, such as the milestone-driven disbursement program of the California's Stem Cell Agency (https://www.cirm.ca.gov/researchers/managing-your-grant#payment). For example, the funding agency could fund the patent and a dose-response study only if animal toxicity studies proved successful. Another model of conditional funding is to require the additional funding from a different source. A funding agency could provide 80% of funding for a project on the condition that the other 20% is covered by a third party. This would be another example of how having an overarching Translational Science Institute could facilitate connections between promising projects and potential funding opportunities. By dividing costs and incorporating intermediate milestones, the risks for funding agencies are kept to a minimum. In addition, translational research, in and of itself, reduces the risk associated with large, long-term projects by internal peer review. Because translational research teams are composed of experts with different backgrounds and skill sets, they can create innovative ideas while simultaneously the individual group members serve as peer-reviewers of their team members and as such many pitfalls will be obviated.

Many private and public funders are now seeking to promote collaboration and network building between academia and industry, incentivizing scientists to “think big” and connect to experts that can help translate their findings (e.g., funding for translational medicine by the NCATS, the Collaborative science award of the American Heart Association, and the private-public-consortium subsidy “Health-Holland”). This new recognition of necessity to actively facilitate translational research pathways has come about through funders' deeper knowledge of their own funding successes and failures, and pressure to be more transparent and accountable to stakeholders and beneficiaries. Taken together, funding agencies are in a position to take the lead to reform the scientific climate and promote translational research and its benefits to society. By developing a grant system specifically for translational research, large funding agencies can promote interdisciplinary research while accommodating long-term timelines inherent to translational research. By staying in dialogue with the scientific community and adjusting funding structure to the current needs, funding agencies will ultimately see a larger return for their investments and a greater impact in improving human health.

## Translational Scientists Require a Multidisciplinary Infrastructure to Succeed

Building an infrastructure to promote education and research in translational medicine is a resource-intensive enterprise. Data suggest that it can show a return on investment (16–18), however such an infrastructure requires a substantial and long-lasting investment of money and time in trainees, mentors and core research facilities. Development and maintenance of adequate shared infrastructures is also considered a major goal for academic centers promoting translational research programs (19, 20). Centralized “hubs” or “cores” that are characterized by shared, multidisciplinary use of expensive laboratory equipment, data power and complex professional skills (e.g., genomics, imaging, flow cytometry, animal facilities, data, and biobanking) are a necessity to maintain institutional competitiveness among universities and research centers around the world. As an example, a central hub named EATRIS (European Research Infrastructure Consortium) was created across Europe to create a proper infrastructure for translational research and it currently includes over 80 top-tier academic institutes (https://eatris.eu). In addition, many universities have started to developed their own infrastructure to support translational research. Examples are valorization offices—tasked with putting academic knowledge to practical use, through offering advice on collaboration with third parties, intellectual property and support for licensing, patenting and entrepreneurship—and a wide variety of incentives for researchers to engage in knowledge transfer activities and focused on the importance of shared biobanking (e.g., BBMRI-ERIC, http://www.bbmri-eric.eu/) and data sharing (e.g., ELIXIR, https://www.elixir-europe.org/) (21).

In similar fashion, hospitals and healthcare providers, tempted by the highly interactive research and clinical care aspects of translational medicine, have started to bring biomedical research and healthcare delivery together inside one highly collaborative space. Case in point, The Shirley Ryan AbilityLab, a rehabilitation hospital born in 2017 from the ashes of the Rehabilitation Institute of Chicago (RIC) is an example where clinical and research laboratories have become the heart of the institution and are both horizontally and vertically integrated and connected to patient care. Another example is the recently opened Princess Maxima Center for Pediatric Oncology in the Netherlands, where an environment is created where research scientists and physicians, clinical personnel, patients and intellectual property officers interact regularly to support new thinking and to promote translational research. The hope is that this milieu, where research is brought to the patient and not the other way around, will lead to the transformation of care with the intent of leveraging the convergence of science, engineering and technology to rapidly advance outcomes.

Financial support for building academic infrastructure and collaborative programs that support translational medicine is traditionally provided through competitive funding programs from public national Agencies. In the US, NIH-sponsored NCATS' Clinical Translational Science Awards (CTSA, https://ncats.nih.gov/ctsa) have provided substantial support for the development of clinical and translational research enterprises through the establishment of research hubs that provide core resources, essential mentoring and training in translational medicine. Examples of such CTSAs are the Institute for Translational Medicine (ITM, https://chicagoitm.org/) and Northwestern University Clinical and Translational Sciences Institute (NUCATS, https://www.nucats.northwestern.edu/). During the formative years of the CTSA program, several sites also forged collaborations by creating regional consortia based on geographic proximity to enable sharing of local resources and meetings of trainees, including the Chicago Consortium for Community Engagement (C3)—a collaboration among Chicago CTSAs—and the Sharing Partnership for Innovative Research in Translation (SPIRiT) Consortium, a model for collaboration across CTSA Sites (22).

## The way Forward to Translational Science

In summary, to create the translational science discipline necessary to rapidly bridge the gap between bench and bedside, highly trained physician scientists and basic scientists that focus on patient-oriented research outcomes are equally needed. To allow talented scientists to develop an identity and career as a translational scientist the current academic system needs to be reformed. Advances in training and recruiting translational scientists in academia will ultimately depend on the research and funding priorities that are set at a national level. For both physician and basic scientists, early exposure to clinically-relevant research and educational programs in a communal interdisciplinary environment that stimulates opportunities for clinical and biological ideas to “cross-pollinate” is absolutely necessary. Having a supportive and well-organized institutional framework, including a dedicated graduate or postgraduate program for translational medicine with accessible mentors suitably trained in translational medicine is crucial. Importantly, career evaluation and promotions, and funding opportunities that are designed to match the unique and complex infrastructure of translational science are indispensable for translational scientists to succeed.

## Author Contributions

SvD, AD, NG-O, PH, CL, VM, AvR, and NR developed the concept and outline of the manuscript and contributed to the preparation of the manuscript. SvD and NR with significant help of AD, did the final editing of the manuscript.

### Conflict of Interest Statement

The authors declare that the research was conducted in the absence of any commercial or financial relationships that could be construed as a potential conflict of interest.
